# Computational Image Analysis Identifies Histopathological Image Features Associated With Somatic Mutations and Patient Survival in Gastric Adenocarcinoma

**DOI:** 10.3389/fonc.2021.623382

**Published:** 2021-03-31

**Authors:** Jun Cheng, Yuting Liu, Wei Huang, Wenhui Hong, Lingling Wang, Xiaohui Zhan, Zhi Han, Dong Ni, Kun Huang, Jie Zhang

**Affiliations:** ^1^ National-Regional Key Technology Engineering Laboratory for Medical Ultrasound, Shenzhen University, Shenzhen, China; ^2^ Guangdong Key Laboratory for Biomedical Measurements and Ultrasound Imaging, School of Biomedical Engineering, Health Science Center, Shenzhen University, Shenzhen, China; ^3^ Marshall Laboratory of Biomedical Engineering Shenzhen University, Shenzhen, China; ^4^ Department of Radiation Oncology, Guangdong Provincial People’s Hospital, Guangdong Academy of Medical Sciences, School of Medicine, South China University of Technology, Guangzhou, China; ^5^ School of Basic Medicine, Chongqing Medical University, Chongqin, China; ^6^ Department of Medicine, Indiana University, School of Medicine, Indianapolis, IN, United States; ^7^ Department of Medical and Molecular Genetics, Indiana University School of Medicine, Indianapolis, IN, United States

**Keywords:** computational pathology, gastric adenocarcinoma, gastric cancer, whole-slide image, genotype-phenotype association, prognosis

## Abstract

Computational analysis of histopathological images can identify sub-visual objective image features that may not be visually distinguishable by human eyes, and hence provides better modeling of disease phenotypes. This study aims to investigate whether specific image features are associated with somatic mutations and patient survival in gastric adenocarcinoma (sample size = 310). An automated image analysis pipeline was developed to extract quantitative morphological features from H&E stained whole-slide images. We found that four frequently somatically mutated genes (TP53, ARID1A, OBSCN, and PIK3CA) were significantly associated with tumor morphological changes. A prognostic model built on the image features significantly stratified patients into low-risk and high-risk groups (log-rank test p-value = 2.6e-4). Multivariable Cox regression showed the model predicted risk index was an additional prognostic factor besides tumor grade and stage. Gene ontology enrichment analysis showed that the genes whose expressions mostly correlated with the contributing features in the prognostic model were enriched on biological processes such as cell cycle and muscle contraction. These results demonstrate that histopathological image features can reflect underlying somatic mutations and identify high-risk patients that may benefit from more precise treatment regimens. Both the image features and pipeline are highly interpretable to enable translational applications.

## Introduction

Gastric cancer is the world’s third leading cause of cancer mortality following lung and colorectal cancers (1). Among all gastric cancer, 90% are gastric adenocarcinomas (2). Most patients with gastric cancer are in the late stage at the time of diagnosis, and the tumor has often spread to lymph nodes or other organs; therefore, the prognosis of gastric cancer is usually poor. Stratification of cancer patients into groups with different tumor genotypes, phenotypes and clinical outcomes is a key process to achieve precision oncological treatment. While pathologists’ evaluation of tumor tissue slides remains the gold standard for cancer diagnosis and grading, quantitative biomarkers by computational histopathological image analysis and molecular and genetic tests are rapidly developing and have become more and more important in disease diagnosis and prognosis (3–7).

In clinical practice, the most commonly used indicator for assessing the extent of cancer spreading is tumor node metastasis (TNM) staging (8). TNM staging has been widely used on solid tumors for estimating prognosis and planning treatment for patients. Besides TNM staging, there are alternative classification systems for gastric cancers. For instance, the Lauren classification categorizes gastric cancers into intestinal and diffuse types (9), while World Health Organization proposes to classify gastric cancer into four major histologic patterns: tubular, papillary, mucinous, and poorly cohesive (including signet ring cell carcinoma), plus uncommon histologic variants (10). However, the effectiveness of these classification systems is limited due to the high heterogeneity of gastric cancers. More recently, extensive efforts have been made to take advantage of high-throughput large-scale molecular profiling data in the hope of discovering better diagnostic (11–13), prognostic (14–16), and predictive (17–20) biomarkers.

Besides molecular profiling, the advance in imaging scanning technique combined with machine learning research for feature extraction, has made the analysis on digitized pathology images a very active area of research [for reviews please see (21, 22)]. The resulted computational pathology technologies enable systematic characterization and quantification of the diverse structures present in histopathological images, such as H&E and immunohistochemistry stained images. The extraction of certain quantitative features can capture subtle image differences between phenotypes, some of which are too subtle to be perceived by human eyes and may carry valuable information related to diagnosis and clinical outcomes. A number of studies have been conducted in this direction with applications in cancer research, especially in lung cancers and breast cancers for their high mortality or incidence. These studies include detection and segmentation of nuclei (23–25) and glands (26), tissue classification (27–29), automated cancer diagnosis (30) and prognosis (7, 31).

Since cancer can be characterized by tissue and cellular morphological features from histopathological images and by molecular features from molecular omics data, an interesting scientific question arises — will tumor genetic changes be reflected at the tissue morphological level? Integrative analysis of multimodal data has been previously carried out in cancers such as glioblastoma (32), kidney cancer (3, 6), lung cancer (33), breast cancer (34, 35), and ovarian cancer (36). Using glioblastomas as an example, Cooper et al. demonstrated how computational image analysis can be used to study the relation of the tumor microenvironment to genomic alterations and gene expression patterns and to identify imaging biomarkers predictive of clinical outcomes (32). To the best of our knowledge, there are no such studies conducted for gastric cancer.

In this study, we used a large, multicenter collection of whole-slide images of gastric adenocarcinoma from The Cancer Genome Atlas (TCGA) and extracted quantitative tumor cellular and tissue morphological features. We aimed to perform three tasks with the quantitative image features (1): comparing image features with tumor somatic mutation status (2), evaluating the power of a prognostic model built on these features for predicting patient overall survival, and (3) integrating contributing image features in the prognostic model with gene expression data to find relevant biological processes that may contribute to the tissue morphological differences.

## Materials and Methods

### Data Collection

The TCGA gastric adenocarcinoma (TCGA-STAD project) dataset was used in this study. Four types of data were collected: H&E stained whole-slide images, gene-level somatic mutation consisting of single nucleotide variation (SNVs) and insertions and deletions (indels), gene expression as RNA-seq, and clinical survival data. The whole-slide images were downloaded from Genomic Data Commons portal (https://portal.gdc.cancer.gov/), and the rest were downloaded from University of California Santa Cruz Xena portal (https://xenabrowser.net/datapages/). Only primary tumor samples (indicated by the 14-15th TCGA sample code, 01) were used. The whole-slide images were captured using objective lens with x20 or x40 magnification, resulting in image resolution of ~0.5 µm/pixel (x20) or ~0.25 µm/pixel (x40). The somatic mutation data took the form of binary gene-level non-silent mutation calls: 1 for non-silent mutation and 0 for wild type. Intersection of the four types of data resulted in 310 patients with all four data types available. The demographic and clinical characteristics of the 310 patients are summarized in **Table 1**.

**Table 1:** Patient demographic and clinical characteristics.

Characteristics	Summary
No. of patients	310
Age (year)	
Range	34 – 90
Median	66
Gender	
Female	112 (36.13%)
Male	198 (63.87%)
Follow-up (month)	
Range	0.1 – 124
Median	14.77
Death	116 (37.42%)
TNM staging	
Stage I	43 (13.87%)
Stage II	104 (33.55%)
Stage III	134 (43.23%)
Stage IV	29 (9.35%)
Tumor grade	
G1	5
G2	105
G3	200

### Analysis Workflow

**Figure 1:**
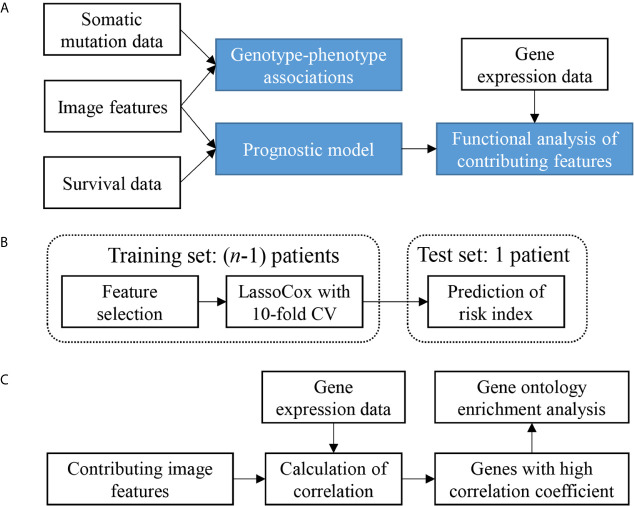
Study design and analysis overview. **(A)** Analysis workflow depicting the overall study design. **(B)** Cross validation scheme showing one round of leave-one-out cross validation. n is the sample size. CV, cross-validation. **(C)** Scheme of the gene functional enrichment analysis of genes that correlated with the contributing features that were frequently selected in the process of leave-one-out cross validation.

The overview of our data analysis workflow design is shown in **Figure 1A**. It consists of three steps. First, genotype-phenotype associations were investigated by comparing image features with somatic mutation status. Then, a prognostic model was constructed based on image features. Finally, a gene functional analysis was conducted for contributing features in the model by integrating image features with gene expression data to identify feature-correlated genes expressions and enriched biological processes which may contribute to the morphological changes.

### Extraction of Quantitative Features From Whole-Slide Images

In view of the color variations between different institutions, before feature extraction we normalized the color appearance of the images using a structure-preserving color normalization algorithm (37). We manually labeled tumor and non-tumor regions in whole-slide images and extracted a total of 150 patient-level image features within the tumor regions using a histopathological image analysis pipeline we previously developed in our lab (38). The feature extraction pipeline is comprised of three steps: segmenting cell nuclei, extracting cell-level features, and summarizing cell-level features into patient-level features. (a) A hierarchical multilevel thresholding method was employed to segment cell nuclei (39). Tissue folds, which are considered as a type of tissue image artifacts, exist in a few images. To avoid the negative impact of tissue folds on the extraction of cellular morphological features, the detected nuclei with very large size (above the 95th percentile calculated in a sample image) were considered as false detections and were excluded from subsequent analyses. (b) For each segmented nucleus, 10 cell-level features were extracted, which characterize nucleus size, shape, texture, and distances to neighboring nuclei. Specifically, these features are nuclear area (denoted by area), lengths of the major and minor axes of the ellipse fitted on the segmented nucleus (denoted by major and minor), the ratio of major axis length to minor axis length (denoted by ratio), mean pixel values of the nucleus in RGB channels respectively (denoted by rMean, gMean, and bMean), and mean, maximal, minimal distances to neighboring nuclei (denoted by distMean, distMax, and distMin). The neighboring relationship of the cells was obtained by constructing a Delaunay triangulation graph based on the centroids of segmented nuclei. In the graph, nuclei were nodes and connected to neighboring nuclei. Therefore, for each nucleus we can calculate its mean, maximal, and minimal distances to its neighbors. (c) For each cell-level feature, we summarized all of them for the images from the same patient into a 10-bin histogram plus five distribution statistics (mean, standard deviation, skewness, kurtosis, and entropy). An individual patient may have multiple slides, and in this case, all slides per patient were treated as a single large slide. It should be noted that the same bin centers were used for all patients to construct histograms, so these histogram features are comparable across patients. Using the cell-level feature area as an example, the corresponding 15 patient-level features were denoted by area_bin1 to area_bin10 for the 10 histogram features; and area_mean, area_std, area_skewness, area_kurtosis, and area_entropy for the five distribution statistics. The patient-level features for other cell-level features were named with the same rule (e.g., ratio_bin1 and ratio_mean are patient-level features for the cell-level feature ratio). **Table S1** provides a list of all the 150 image features as well as their explanations.

Image histogram features allow us to explore the proportion of cells in a tissue slide that fall into specific bins, which may capture the image property of specific cell type or cell subpopulation. For instance, the features ratio_bin1 to ratio_bin10 characterize the shape of cells; therefore, lymphocytes, which are rounder compared with tumor and stromal cells, are more likely to fall into the first few bins. Tumor cells tend to fall into middle bins while spindle-shaped stromal cells such as fibroblasts mostly fall into the last few bins. In addition, the density related features (e.g., distMean, the mean distance of a nucleus to its neighbors) cover the organization of the cells instead of cell nuclei. While the tumor/epithelial cells and infiltrating lymphocytes are more densely packed, the stromal cells such as fibroblasts are more scattered around. Therefore, features such as the first few bins of the histogram of distMean are generally associated with epithelium, while the last few bins of the histogram of distMean are associated with stroma in the tumor microenvironment. Thus, the features extracted from our pipeline not only reflect the tumor cell nuclei morphology, but also quantitatively describe the surrounding texture features which are indicative of microenvironment changes. In contrast to histogram features targeting specific groups of cells, the five statistics measure the distribution of cell-level features of all cells in an image.

### Development of Prognostic Model Based on Histopathological Image Features

To reduce the dimensionality of above feature set, log-rank test was used to filter out noninformative features. Specifically, each feature was dichotomized by its median, and log-rank test was performed to test whether there was significant difference in survival between the two groups. Only features with a p-value less than 0.05 were retained. Since we are in the process of feature screening, we did not apply multiple test compensation at this step in order to keep the trending features. Note that we only used the training set for the initial feature filtering. Then, lassoCox models were trained based on the selected features. LassoCox models were trained using the training set with different regularization parameters, and a 10-fold cross-validation was used to select the best parameter. The model with the optimal regularization parameter was applied to the test set to calculate the risk index for each patient.

The scheme for the risk index calculation for each patient is shown in **Figure 1B**. LOOCV was used in this study. For each round of LOOCV, a single patient was selected as the test set and the remaining were used as the training set. This process was repeated *n* times (*n* is the sample size) until all patients were tested. After *n* rounds, each patient was assigned with a risk index, and then all patients were classified into either the low-risk or high-risk group using the median risk index as a cutoff. Note that the above feature selection, model training, and model selection processes were all performed only in the training set to avoid mixing with the testing sample. The R package glmnet was used for training lassoCox model.

### Discovery of the Biological Processes Related to the Contributing Features in the Prognostic Model

In the process of LOOCV, we constructed *n* lassoCox models (*n* is the sample size). Each model is a linear combination of features selected by lasso, weighted by the corresponding non-zero coefficients. The selection frequency and the corresponding mean coefficient of each feature indicate its robustness and contribution to the risk index. How to interpret these contributing features biologically is a question of our particular interest. Here we tried to identify the biological processes related to the contributing features by an integrative analysis of histopathological image features with gene expression data (**Figure 1C**). Specifically, for each of the most frequently selected features, we calculated the Spearman’s correlations between each of the features and the gene expression over the corresponding patients, then selected the top genes with relatively high correlation (|Spearman’s correlation coefficient| ≥ 0.3), and performed GO enrichment analysis based on the obtained gene set.

### Statistical Analysis

A two-sided Mann-Whitney U test was used to compare image feature distributions between mutated and nonmutated cases for each gene. False discovery rate (FDR) adjustment was applied for multiple testing correction (40). The FDR adjusted p-value was also denoted by q-value for notational convenience. The Kaplan-Meier method was used to estimate the survival rate, and the log-rank test was performed to test survival difference between different groups. Univariable and multivariable Cox proportional hazards regression models were used to investigate the additional prognostic value of the lassoCox model over multiple clinical factors. Three clinical factors were considered: tumor stage, tumor grade, and MSI status. All survival analyses were performed using the R package survival. Functional enrichment analysis of gene set was performed using the online tool ToppGene (41) with all parameters in default settings. Tests were considered statistically significant if q-value < 0.05, or p-value < 0.05 was used where FDR adjustment is unnecessary.

## Results

### Gene Mutations Are Associated With Tissue Morphological Changes

**Figure 2:**
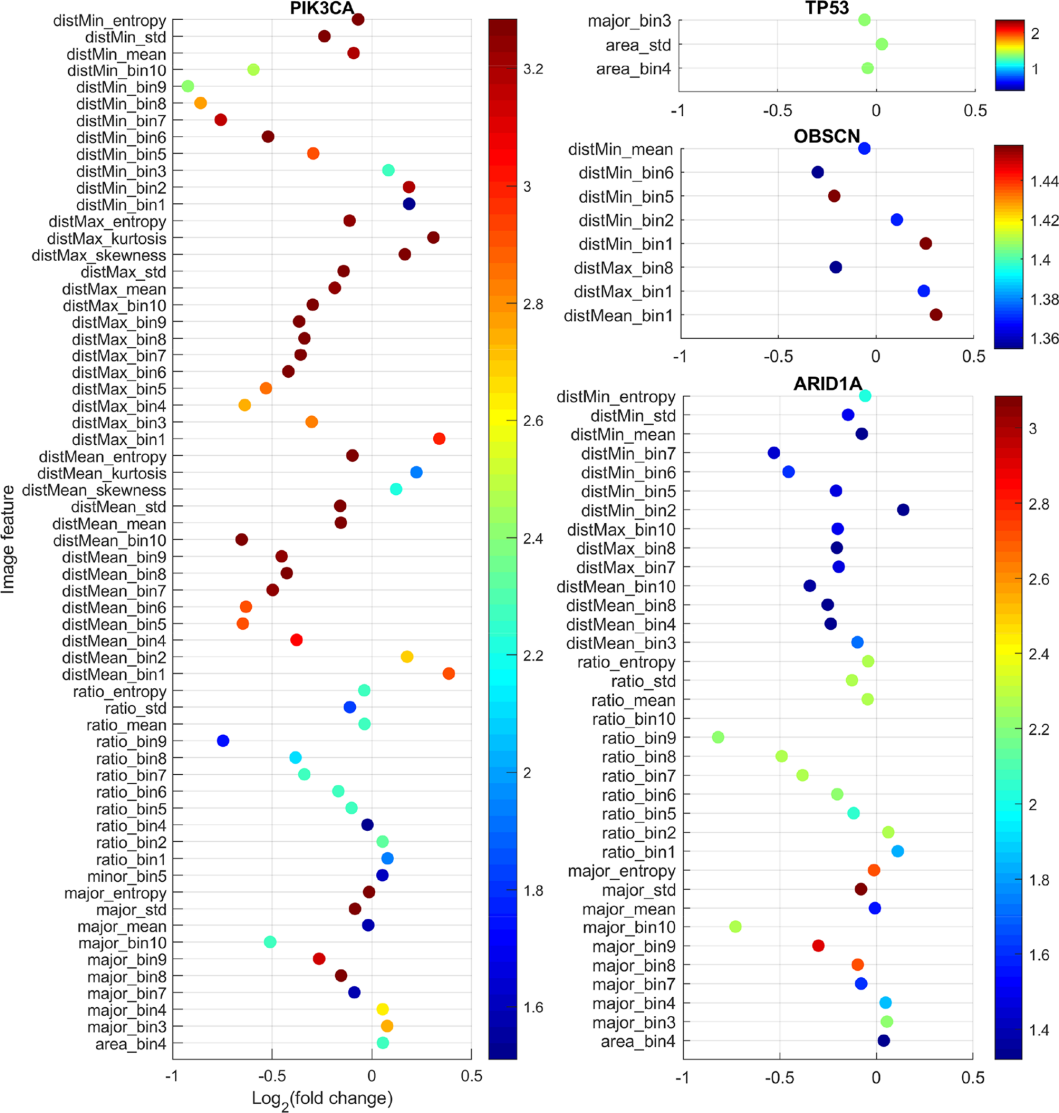
Comparison of image features with respect to somatic mutation status. For each feature, the fold change is the ratio of the median feature values between the mutated group and nonmutated group (mutated/nonmutated), and the color scale is the negative logarithm of q-value. The dots’ color corresponds to the scales of the side color bars.

With the curation of large-scale genomic data, genes with mutations that are common to gastric adenocarcinoma have been identified. In this study, 14 genes with somatic mutations (non-synonymous SNVs and indels) in more than 15% patients in our dataset were identified: *TP53* 49.35%, *TTN* 36.77%, *ARID1A* 25.48%, *MUC16* 24.84%, *LRP1B* 22.58%, *SYNE1* 21.94%, *CSMD1* 20.97%, *CSMD3* 19.35%, *OBSCN* 18.39%, *FLG* 18.39%, *PIK3CA* 16.45%, *SPTA1* 16.13%, *PCDH15* 15.81%, and *FAT4* 15.16%. While previous studies often focused on the effects of mutations on molecular events such as mRNA/microRNA expression and DNA methylation, it is of our great interest to examine the relationship between somatic mutations and the quantifiable morphological changes in tumor tissues. Such relationship will help us understand the impact of the mutated genes on the cell or tissue growth and organization, and therefore provides insights on the morphological distinctions behind different cancer subtypes. We hypothesized that tissue morphological features could reflect underlying genetic mutations. To test this hypothesis, we compared the 150 image features between patients with mutated and nonmutated gene for each of the above listed 14 frequently mutated genes using the Mann-Whitney U test. The results after multiple testing correction showed that four genes (*TP53*, *ARID1A*, *OBSCN*, and *PIK3CA*) were significantly associated with some image features. **Figure 2** shows for each morphological feature the fold change and q-value. The fold change is the ratio between the median value of the specific feature in the mutated patient group and its median value in nonmutated group (mutated/nonmutated). For understanding the image features, please refer to *Extraction of Quantitative Features From Whole-Slide Images* and **Table S1**.

*TP53* is a well-studied tumor suppressor gene, which regulates cell division by keeping cells from growing and proliferating uncontrollably. Mutation in this gene often causes cancer. As expected, we found that the mutation status of *TP53* gene was significantly associated with image features characterizing cell size such as area_std and area_bin4. The feature area_std is the standard deviation of nucleus size in an image. As we can see in **Figure 2**, the fold change for area_std was greater than one. This means that compared with *TP53* wild-type tumor samples, tumor samples with *TP53* mutations tend to have more heterogeneous cell size, which is consistent with the function of the *TP53* gene being a cell growth controller.

*ARID1A* encodes a member of the SWI/SNF family, whose members are thought to regulate gene expression by chromatin remodeling (42). Through their ability to regulate gene activity, SWI/SNF complexes are involved in many processes including DNA damage repair; and controlling the growth, division, and differentiation of cells. Arid1a protein and other SWI/SNF subunits are thought to act as tumor suppressors. *ARID1A* mutation status was significantly associated with image features such as major_bin10, major_bin9, ratio_bin10, ratio_bin9, and distMean_bin10 (**Figure 2**). These features characterize the proportion of stromal cells and cell density in tumors. Stromal cells such as fibroblasts are typically spindle-shaped with elongated nuclei and play an important role in the tumor microenvironment to support tumor growth. ratio_bin10 and ratio_bin9 reflect the proportions of cells with highly elongated nuclei. These features’ fold changes were less than one. Similarly, disMean_bin10 and distMean_bin8, both of which reflect the proportions of nuclei with large distance to their neighbors, their fold changes were also less than one. All these features indicate that tumor samples with *ARID1A* mutations have significantly denser cells and less stroma, which is consistent with the function of *ARID1A* as a tumor suppressor.

*OBSCN* encodes a protein called obscurin. Obscurin is a member of the family of giant sarcomeric signaling proteins that includes titin and nebutin. Somatic mutation status of this gene is significantly associated with eight features that are all related to cell distance (**Figure 2**). For example, the cell-level feature distMin is the distance of a nucleus to its closest neighbor. distMin_bin1 and distMin_bin2 represent the proportion of nuclei whose distance to their closest neighbor is very short implying dense cell clumping, whereas distMin_bin5 and distMin_bin6 denote the proportion of nuclei with the distance to their closest neighbor being medium to relatively long. Our analysis showed that distMin_bin1 and distMin_bin2 had fold changes greater than 1 while distMin_bin5 and distMin_bin6 had fold changes less than 1, which indicate that *OBSCN* mutant tumors tend to generate more cell clumps than wild-type tumors. The finding that this protein affects the cell and tissue level morphology is a potential novel discovery.

*PIK3CA* gene encodes p110 alpha protein, which is a subunit of an enzyme phosphatidylinositol 3-kinase (PI3K). PI3K signaling is important for many cell activities, such as cell growth and proliferation, cell migration, and protein production. *PIK3CA* mutation status was significantly associated with 62 image features. Among them, features such as distMean_bin10, distMean_mean, ratio_bin9, and major_bin10 showed significant decreases in the *PIK3CA* mutant tumors, while features like distMean_bin1, distMean_bin2, ratio_bin1, and ratio_bin2 showed significant increases in the mutant group, indicating denser cells and less stroma for *PIK3CA* mutant tumors. This association is consistent with the role of *PIK3CA* gene as an oncogene (43).

### An Effective Model for Predicting Gastric Adenocarcinoma Prognosis

**Figure 3:**
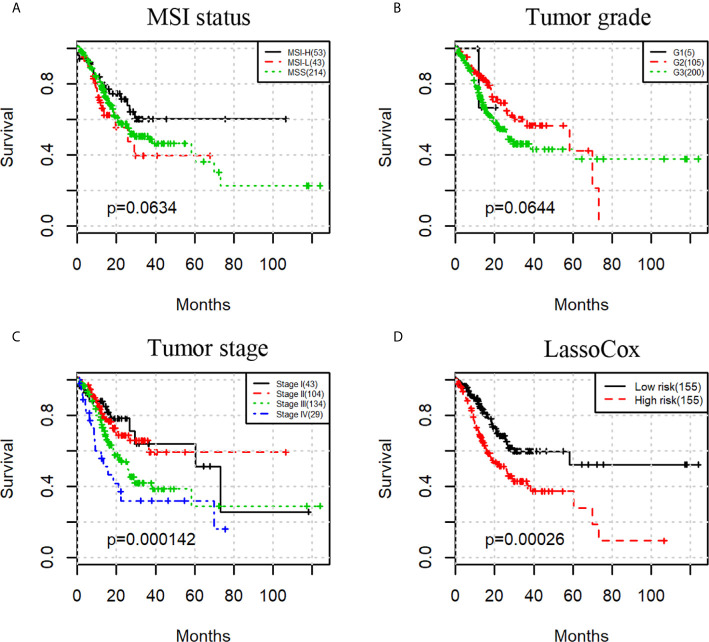
Kaplan-Meier survival curves for patients stratified by MSI status **(A)**, tumor grade **(B)**, tumor stage **(C)**, and lassoCox **(D)**.

We then investigated whether a prognostic model built on selected image features can predict the overall survival of the gastric adenocarcinoma patients. As a comparison, we first evaluated the prognostic power of three commonly used clinical factors, that is, microsatellite instability (MSI) status, tumor grade, and tumor stage. There was no significant difference in overall survival when patients were split by MSI status or tumor grade with the uncorrected log-rank p-values being 0.0634 and 0.0644 respectively (**Figures 3A, B**). However, tumor stage stratified patients with significant survival difference with a log-rank test p-value less than 0.001 (**Figure 3C**).

Based on the image features identified from each round of model training, lasso-regularized Cox proportional hazards (lassoCox for short) models were developed to predict the risk index for each patient, and then patients were split into two groups: low-risk group and high-risk group (see *Development of Prognostic Model Based on Histopathological Image Features*). As expected, patients in the high-risk group had much lower survival rate than those in the low-risk group (log-rank test p-value = 0.00026; **Figure 3D**). We further investigated whether our prognostic model built on selected image features had additional prognostic value over clinical factors using univariable and multivariable Cox proportional hazards regression models. Tumor stage, tumor grade, and MSI status were dichotomized as follows: stage I + II vs stage III + IV, G1 + G2 vs G3, and MSS (microsatellite stable) vs MSI-L (MSI-low) + MSI-H (MSI-high). For each factor, the first group was considered to be the reference group. As we can see in **Table 2**, MSI status and tumor grade were not significant in the univariable or multivariable model, though tumor grade was bordering on the statistical significance in the univariable model. In contrast, lassoCox and tumor stage were strongly associated with survival in both univariable and multivariable model, suggesting that the lassoCox model derived from the image features has independent prognostic value.

**Table 2:** Univariable and multivariable Cox proportional hazards model analysis of the prognostic value of lassoCox and three clinical factors.

	Univariable analysis		Multivariable analysis	
	HR (95% CI)	p-value	HR (95% CI)	p-value
LassoCox: high-risk (vs low-risk)	2.00 (1.37-2.92)	3.44e-4	2.04 (1.39-3.00)	2.64e-4
Tumor stage: III+IV (vs I+II)	1.92 (1.30-2.82)	9.53e-4	1.95 (1.32-2.88)	7.73e-4
Tumor grade: G3 (vs G1+G2)	1.46 (0.98-2.17)	0.0662	1.27 (0.85-1.91)	0.248
MSI status: MSI-L+MSI-H (vs MSS)	0.89 (0.60-1.34)	0.590	0.95 (0.63-1.42)	0.800

The group in parenthesis is the reference group. HR, hazard ratio; CI, confidence interval.

### Functional Enrichment Analysis of the Contributing Features in the Prognostic Model

In the process of leave-one-out cross validation (LOOCV), we found that five image features were selected more than 80% times, indicating the importance and robustness of these features for prognosis. The five features and their mean coefficients from the multiple lassoCox models were listed in **Table 3**. We calculated the Spearman’s correlations between the five image features and gene expression data, and performed gene ontology (GO) enrichment analysis for each feature based on the top 100 genes whose correlation coefficients were greater than 0.3 either positively or negatively, if available. As a result, three image features meet this criterion and are shown in **Table 3**. The other two features only resulted in one or two genes with correlation coefficients greater than 0.3, so GO enrichment analysis was not performed for these two features.

**Table 3:** The biological processes of the top five most frequently selected image features.

Image feature	Mean coefficient	Count of selection/sample size	Enriched biological process for correlated genes
bMean_bin6	0.209	310/310	N/A
distMin_bin5	0.250	310/310	Cell cycle (GO:0007049)Cell cycle process (GO:0022402)Organelle fission (GO:0048285)
ratio_skewness	0.311	310/310	Muscle contraction (GO:0006936)Muscle system process (GO:0003012)Regulation of muscle contraction (GO:0006937)
major_bin10	-0.124	283/310	Muscle contraction (GO:0006936)Muscle system process (GO:0003012)Regulation of muscle contraction (GO:0006937)
distMean_bin3	0.050	268/310	N/A

Note for each round of LOOCV, features were selected independently, so some features may not be selected every time (for example, major_bin10 and distMean_bin3).

As we can see in **Table 3**, except major_bin10, the other four features (bMean_bin6, distMin_bin5, ratio_skewness, and distMean_bin3) all had positive mean coefficients, meaning that the higher the feature value the higher the survival risk. bMean_bin6 is the proportion of the nuclei that have medium level of pixel intensity in the blue channel. It was most highly correlated with the *TMEM109* gene, whose protein product mediates cellular response to DNA damage by protecting against ultraviolet C-induced cell death (44). distMin_bin5 is the proportion of nuclei whose distance to their closest neighbors is of medium length. GO enrichment analysis showed that the genes correlated with distMin_bin5 were related to cell cycle, cell cycle process, and organelle fission, suggesting that cell cycle and organelle fission processes may play roles in determining the density of the nuclei. ratio_skewness and major_bin10 are related to nucleus shape. ratio_skewness is a measure of the asymmetry of the distribution of ratio between nucleus major and minor axis length. major_bin10 is the proportion of the nuclei that have a very long major axis length. The most enriched GO biological processes for ratio_skewness and major_bin10 were muscle contraction, muscle system process, and regulation of muscle contraction, which indicates that the processes that contributes to muscle cell function and development may also contribute to the nuclei shape elongation. distMean_bin3 is the proportion of nuclei with very close neighbors. The most correlated gene with this feature is *F11R*, which encodes an important protein regulating tight junction assembly in epithelial cells. Therefore, it is not surprising that this gene expression correlates with the proportion of nuclei whose distance to their closest neighbors is of less than medium length (distMean_bin3), i.e., a tight arrangement of the cells.

## Discussion

The TCGA project not only hosts a large collection of genomic and clinical data for 33 cancer types, but also provides a large collection of H&E stained histopathology images for 32 types of solid tumors. Although the TCGA research team and other groups have conducted comprehensive analyses on genomic, epigenomic, transcriptomic, and proteomic data, the rich and valuable information in the digital imaging archive remains largely underutilized. Notably, a recently published work generated the tumor-infiltrating lymphocyte maps of 5,202 digital whole-slide images from 13 cancer types, demonstrating one example of using this rich image archive with insights into the tumor-immune microenvironment (27). Here, to further promote the extensive utilization of this valuable imaging resource, we conducted an integrative analysis on imaging, somatic mutation, gene expression, and clinical data for the less-studied gastric adenocarcinoma. We developed an automated image analysis pipeline to extract a large set of morphological features from whole-slide images, and then investigated their associations with tumor somatic mutation and patient overall survival. The image features contributing significantly to the prognostic model were further investigated for their relationship with gene expression data to analyze potentially relevant biological processes.

In this study, we found that the somatic mutation status of *TP53*, *ARID1A*, *OBSCN*, and *PIK3CA* was significantly associated with tumor tissue morphological changes, which confirms our hypothesis that quantitative tissue morphological features can reflect underlying genetic mutations, and their association with the tissue level morphology is a novel finding. TCGA and PanCancer Initiative have identified genetic alterations such as gene mutations present across multiple cancer types (45). Some of these alterations may serve as potential therapeutic targets, such as mutant *ARID1A*, *BARF*, and *EGFR* (46). According to a recent study (47), loss-of-function mutations in the chromatin remodeling gene *ARID1A* disrupt DNA mismatch repair (MMR) and improve outcomes for mice treated with PD-1-targeting immune checkpoint inhibition. One of the mechanisms involved may be that the accumulating neoantigens over time due to MMR deficiency increase tumor appearance to immune cells. In this study, our results showed that *ARID1A* mutation was linked to significant change of tumor morphology (denser cells and less stroma), suggesting that image features could be potential predictive biomarkers of immunotherapy response.

A major advantage of imaging biomarkers is the ubiquitous availability of H&E slides; they are available for virtually every cancer patient. Furthermore, with the histopathological image features affected by these genes that may mediate highly aggressive disease progression, histopathological image features can serve as pre-screening biomarkers for the patients with those somatic mutations, and an easy tumor image screening can quickly spot these candidates for further genetic sequencing.

More importantly, the identified image features can be combined and serve as a powerful prognostic biomarker. The risk index derived from the image features could stratify patients into two groups with significantly different overall survival, outperforming the tumor grade and MSI, and is independent of clinical factors such as tumor grade and stage. Tumor grading is also based on microscopic morphology of a tumor with H&E staining and describes the extent to which a tumor resembles its tissue of origin. Poorly differentiated tumors (high tumor grade), which have abnormal-looking cells, tend to be more aggressive than well differentiated ones; however, tumor grade as a feature, its association with survival was not significant, as shown in **Figure 3B**. This may be due to the discordance and grading imprecision from subjective histopathological evaluation. In contrast to pathologist’s manual evaluation, our method can generate objective and reproducible features from H&E images using an automated image analysis pipeline. Tumor stage, as expected, is a strong prognosticator. In a multivariable analysis model, both tumor stage and our lassoCox were significantly correlated with survival, indicating that the lassoCox complements tumor stage and that they can be used in combination to provide better patient stratification.

In summary, we demonstrate the power of computational analysis of histopathological images for investigating genotype-phenotype associations and discovering novel imaging prognostic biomarkers. The study of genotype-phenotype associations shows great promise for understating the underlying molecular bases of tumor morphology. Considering the wide availability of tissue slides and adoption of digital scanner, our approach can be easily incorporated into existing clinical workflows to help clinician identify high-risk patients who might benefit from more precisely designed treatment regimens.

## Data Availability Statement

Publicly available datasets were analyzed in this study. This data can be found here: Whole-slide images are available at Genomic Data Commons portal (https://portal.gdc.cancer.gov/repository). Under the “Files” tab, select “Tissue Slide” for “Experimental Strategy”, and under the “Cases” tab, select “TCGA-STAD” for “Project”. Survival information (phenotype, curated survival data), gene-level somatic mutation data, and gene expression RNAseq data (IlluminaHiSeq pancan normalized) are available at University of California Santa Cruz Xena portal (https://xenabrowser.net/datapages/, select TCGA Stomach Cancer).

## Ethics Statement

The studies involving human participants were reviewed and approved by The Cancer Genome Atlas. The patients/participants provided their written informed consent to participate in this study.

## Author Contributions

JC, DN, KH, and JZ conceived the study. YL, WH, WHH, and LW collected and preprocessed the data. JC, XZ, and ZH performed feature extraction and analyzed and interpreted data. JC wrote the manuscript. DN, KH, and JZ supervised the project and revised the manuscript. All authors contributed to the article and approved the submitted version.

## Funding

This study was supported in part in by National Natural Science Foundation of China (No. 61901275 to JC), Shenzhen University startup fund (No. 2019131 to JC), Young Faculty Support Program of SZU Health Science Center (No. 71201000001 to JC), National Key R&D Program of China (No. 2019YFC0118300 to DN), Shenzhen Peacock Plan (No. KQTD2016053112051497 and KQJSCX20180328095606003 to DN), Medical Scientific Research Foundation of Guangdong Province, China (No. B2018031 to DN), American Cancer Society Institutional Research Grant to Indiana University to JZ, and Indiana University Precision Health Initiative to KH and JZ.

## Conflict of Interest

The authors declare that the research was conducted in the absence of any commercial or financial relationships that could be construed as a potential conflict of interest.

## References

[1] Bray F Ferlay J Soerjomataram I Siegel R Torre L Jemal A. Global cancer statistics 2018: GLOBOCAN estimates of incidence and mortality worldwide for 36 cancers in 185 countries. CA A J Clin (2018) 00:1–31. 10.3322/caac.21492 30207593

[2] Kumar V Abbas AK Fausto N Aster JC. Pathologic basis of disease. Elsevier (2010).

[3] Shao W Huang K Han Z Cheng J Cheng L Wang T. Integrative Analysis of Pathological Images and Multi-Dimensional Genomic Data for Early-Stage Cancer Prognosis. IEEE Trans Med Imaging (2020) 39:99–110. 10.1109/TMI.2019.2920608 31170067

[4] Kather JN Krisam J Charoentong P Luedde T Herpel E Weis CA. Predicting survival from colorectal cancer histology slides using deep learning: A retrospective multicenter study. PloS Med (2019) 16:1–22. 10.1371/journal.pmed.1002730 PMC6345440 30677016

[5] Campanella G Hanna MG Geneslaw L Miraflor A Werneck Krauss Silva V Busam KJ. Clinical-grade computational pathology using weakly supervised deep learning on whole slide images. Nat Med (2019) 25:1301–9. 10.1038/s41591-019-0508-1 PMC7418463 31308507

[6] Cheng J Zhang J Han Y Wang X Ye X Meng Y. Integrative Analysis of Histopathological Images and Genomic Data Predicts Clear Cell Renal Cell Carcinoma Prognosis. Cancer Res (2017) 77:e91–100. 10.1158/0008-5472.CAN-17-0313 29092949 PMC7262576

[7] Cheng J Mo X Wang X Parwani A Feng Q Huang K. Identification of topological features in renal tumor microenvironment associated with patient survival. Bioinformatics (2017) 34:1–7. 10.1093/bioinformatics/btx723 PMC7263397 29136101

[8] Amin MB Greene FL Edge SB Compton CC Gershenwald JE Brookland RK. The Eighth Edition AJCC Cancer Staging Manual: Continuing to build a bridge from a population-based to a more “personalized” approach to cancer staging. CA Cancer J Clin (2017) 67:93–9. 10.3322/caac.21388 28094848

[9] Lauren P. The two histological main types of gastric carcinoma: diffuse and so-called intestinal-type carcinoma. Acta Pathol Microbiol Scand (1965) 64:31–49. 10.1111/apm.1965.64.1.31 14320675

[10] Nagtegaal ID Odze RD Klimstra D Paradis V Rugge M Schirmacher P. The 2019 WHO classification of tumours of the digestive system. Histopathology (2019) 76:182–8. 10.1111/his.13975 PMC7003895 31433515

[11] Lei Z Tan IB Das K Deng N Zouridis H Pattison S. Identification of molecular subtypes of gastric cancer with different responses to pi3-kinase inhibitors and 5-fluorouracil. Gastroenterology (2013) 145:554–65. 10.1053/j.gastro.2013.05.010 23684942

[12] Nam S Lee J Goh S-H Hong S-H Song N Jang S-G. Differential gene expression pattern in early gastric cancer by an integrative systematic approach. Int J Oncol (2012) 41:1675–82. 10.3892/ijo.2012.1621 PMC3982715 22961301

[13] Brettingham-Moore KH Duong CP Heriot AG Thomas RJS Phillips WA. Using gene expression profiling to predict response and prognosis in gastrointestinal cancers-the promise and the perils. Ann Surg Oncol (2011) 18:1484–91. 10.1245/s10434-010-1433-1 21104326

[14] Zhang X Yan Z Zhang J Gong L Li W Cui J. Combination of hsa-miR-375 and hsa-miR-142-5p as a predictor for recurrence risk in gastric cancer patients following surgical resection. Ann Oncol (2011) 22:2257–66. 10.1093/annonc/mdq758 21343377

[15] Xu Z-Y Chen J-S Shu Y-Q. Gene expression profile towards the prediction of patient survival of gastric cancer. BioMed Pharmacother (2010) 64:133–9. 10.1016/j.biopha.2009.06.021 20005068

[16] Kim M Rha SY. Prognostic index reflecting genetic alteration related to disease-free time for gastric cancer patient. Oncol Rep (2009) 22:421–31. 10.3892/or_00000454 19578786

[17] Cheong JH Yang HK Kim H Kim WH Kim YW Kook MC. Predictive test for chemotherapy response in resectable gastric cancer: a multi-cohort, retrospective analysis. Lancet Oncol (2018) 19:629–38. 10.1016/S1470-2045(18)30108-6 29567071

[18] Jung JJ Jeung HC Chung HC Lee JO Kim TS Kim YT. In vitro pharmacogenomic database and chemosensitivity predictive genes in gastric cancer. Genomics (2009) 93:52–61. 10.1016/j.ygeno.2008.08.002 18804159

[19] Murakami H Ito S Tanaka H Kondo E Kodera Y Nakanishi H. Establishment of new intraperitoneal paclitaxel-resistant gastric cancer cell lines and comprehensive gene expression analysis. Anticancer Res (2013) 33:4299–308. 24122996

[20] Amini A Sanati H. Complete pathologic response with combination oxaliplatin and 5-fluorouracil chemotherapy in an older patient with advanced gastric cancer. Anticancer Drugs (2011) 22:1024–6. 10.1097/CAD.0b013e32834a2c16 21822120

[21] Niazi MKK Parwani AV Gurcan MN. Digital pathology and artificial intelligence. Lancet Oncol (2019) 20:e253–61. 10.1016/S1470-2045(19)30154-8 PMC8711251 31044723

[22] Madabhushi A Lee G. Image Analysis and Machine Learning in Digital Pathology: Challenges and Opportunities. Med Image Anal (2016) 33:170–5. 10.1016/j.media.2016.06.037 PMC5556681 27423409

[23] Rojas-Moraleda R Xiong W Halama N Breitkopf-Heinlein K Dooley S Salinas L. Robust detection and segmentation of cell nuclei in biomedical images based on a computational topology framework. Med Image Anal (2017) 38:90–103. 10.1016/j.media.2017.02.009 28314191

[24] Kumar N Verma R Sharma S Bhargava S Vahadane A. Sethi A. A Dataset and a Technique for Generalized Nuclear Segmentation for Computational Pathology. IEEE Trans Med Imaging (2017) 36:1550–60. 10.1109/TMI.2017.2677499 28287963

[25] Sirinukunwattana K Raza SEA Tsang Y Snead DRJ Cree IA Rajpoot NM. Locality Sensitive Deep Learning for Detection and Classification of Nuclei in Routine Colon Cancer Histology Images. IEEE Trans Med Imaging (2016) 0062:1–1. 10.1109/TMI.2016.2525803 26863654

[26] Sirinukunwattana K Pluim JPW Chen H Qi X Heng PA Guo YB. Gland segmentation in colon histology images: The glas challenge contest. Med Image Anal (2017) 35:489–502. 10.1016/j.media.2016.08.008 27614792

[27] Saltz J Gupta R Hou L Kurc T Singh P Nguyen V. Spatial Organization and Molecular Correlation of Tumor-Infiltrating Lymphocytes Using Deep Learning on Pathology Images. Cell Rep (2018) 23:181–93.e7. 10.1016/j.celrep.2018.03.086 29617659 PMC5943714

[28] Huang Y Zheng H Liu C Ding X Rohde G. Epithelium-stroma classification *via* convolutional neural networks and unsupervised domain adaptation in histopathological images. IEEE J BioMed Heal Inf (2017) 14:1–1. 10.1109/JBHI.2017.2691738 28410112

[29] Li G Raza SEA Rajpoot NM. Multi-resolution cell orientation congruence descriptors for epithelium segmentation in endometrial histology images. Med Image Anal (2017) 37:91–100. 10.1016/j.media.2017.01.006 28161567

[30] Coudray N Ocampo PS Sakellaropoulos T Narula N Snuderl M Fenyö D. Classification and mutation prediction from non–small cell lung cancer histopathology images using deep learning. Nat Med (2018) 24:1559–67. 10.1038/s41591-018-0177-5 PMC9847512 30224757

[31] Mobadersany P Yousefi S Amgad M Gutman DA Barnholtz-Sloan JS Velázquez Vega JE. Predicting cancer outcomes from histology and genomics using convolutional networks. Proc Natl Acad Sci (2018) 115:E2970–9. 10.1073/pnas.1717139115 PMC5879673 29531073

[32] Cooper LADD Kong J Gutman DA Dunn WD Nalisnik M Brat DJ. Novel genotype-phenotype associations in human cancers enabled by advanced molecular platforms and computational analysis of whole slide images. Lab Investig (2015) 95:366–76. 10.1038/labinvest.2014.153 PMC4465352 25599536

[33] Wang C Su H Yang L Huang K. Integrative analysis for lung adenocarcinoma predicts morphological features associated with genetic variations. Pac Symp Biocomput (2017) 22:82–93. 10.1142/9789813207813_0009 27896964 PMC5360185

[34] Wang C Pecot T Zynger DL Machiraju R Shapiro CL Huang K. Identifying survival associated morphological features of triple negative breast cancer using multiple datasets. J Am Med Inform Assoc (2013) 20:680–7. 10.1136/amiajnl-2012-001538 PMC3721170 23585272

[35] Zhan X Cheng J Huang Z Han Z Helm B Liu X. Correlation analysis of histopathology and proteogenomics data for breast cancer. Mol Cell Proteomics (2019) 18:S37–51. 10.1074/mcp.RA118.001232 PMC6692775 31285282

[36] Martins FC De SI Trinh A Xian J Guo A Sayal K. Combined image and genomic analysis of high-grade serous ovarian cancer reveals PTEN loss as a common driver event and prognostic classifier. Genome Biol (2014) 15:526. 10.1186/s13059-014-0526-8 25608477 PMC4268857

[37] Vahadane A Peng T Sethi A Albarqouni S Wang L Baust M. Structure-Preserving Color Normalization and Sparse Stain Separation for Histological Images. IEEE Trans Med Imaging (2016) 0062:1–1. 10.1109/TMI.2016.2529665 27164577

[38] Cheng J Han Z Mehra R Shao W Cheng M Feng Q. Computational analysis of pathological images enables a better diagnosis of TFE3 Xp11.2 translocation renal cell carcinoma. Nat Commun (2020) 11:1–9. 10.1038/s41467-020-15671-5 32286325 PMC7156652

[39] Ahmady Phoulady H Goldgof DB Hall LO Mouton PR. Nucleus segmentation in histology images with hierarchical multilevel thresholding. Proc SPIE 9791 Med Imaging 2016 Digit Pathol (2016) 9791:979111. 10.1117/12.2216632

[40] Yekutieli D Benjamini Y. Resampling-based false discovery rate controlling multiple test procedures for correlated test statistics. J Stat Plan Inference (1999) 82:171–96. 10.1016/S0378-3758(99)00041-5

[41] Chen J Bardes EE Aronow BJ Jegga AG. ToppGene Suite for gene list enrichment analysis and candidate gene prioritization. Nucleic Acids Res (2009) 37:W305–11. 10.1093/nar/gkp427 PMC2703978 19465376

[42] Jones S Li M Williams Parsons D Zhang X Wesseling J Kristel P. Somatic mutations in the chromatin remodeling gene ARID1A occur in several tumor types. Hum Mutat (2012) 33:100–3. 10.1002/humu.21633 PMC3240719 22009941

[43] Karakas B Bachman KE Park BH. Mutation of the PIK3CA oncogene in human cancers. Br J Cancer (2006) 94:455–9. 10.1038/sj.bjc.6602970 PMC2361173 16449998

[44] Yamazaki T Sasaki N Nishi M Takeshima H. Facilitation of DNA damage-induced apoptosis by endoplasmic reticulum protein mitsugumin23. Biochem Biophys Res Commun (2010) 392:196–200. 10.1016/j.bbrc.2010.01.013 20060811

[45] Kandoth C McLellan MD Vandin F Ye K Niu B Lu C. Mutational landscape and significance across 12 major cancer types. Nature (2013) 503:333–9. 10.1007/s13398-014-0173-7.2 PMC3927368 24132290

[46] Cooper LA Demicco EG Saltz JH Powell RT Rao A Lazar AJ. PanCancer insights from The Cancer Genome Atlas: The pathologist’s perspective. J Pathol (2018) 244:512–24. 10.1002/path.5028 PMC6240356 29288495

[47] Shen J Ju Z Zhao W Wang L Peng Y Ge Z. ARID1A deficiency promotes mutability and potentiates therapeutic antitumor immunity unleashed by immune checkpoint blockade. Nat Med (2018) 24:556–62. 10.1038/s41591-018-0012-z PMC6076433 29736026

